# Complex association of apolipoprotein E–containing HDL with coronary artery disease burden in cardiovascular disease

**DOI:** 10.1172/jci.insight.159577

**Published:** 2022-05-23

**Authors:** Alexander V. Sorokin, Nidhi Patel, Khaled M. Abdelrahman, Clarence Ling, Mart Reimund, Giorgio Graziano, Maureen Sampson, Martin P. Playford, Amit K. Dey, Aarthi Reddy, Heather L. Teague, Michael Stagliano, Marcelo Amar, Marcus Y. Chen, Nehal N. Mehta, Alan T. Remaley

**Affiliations:** 1 Section of Lipoprotein Metabolism, Translational Vascular Medicine Branch, and; 2 Section of Inflammation and Cardiometabolic Diseases, Cardiovascular Branch, National Heart, Lung, and Blood Institute (NHLBI), National Institutes of Health (NIH), Bethesda, Maryland, USA.

**Keywords:** Cardiology, Metabolism, Cardiovascular disease, Diagnostic imaging, Lipoproteins

## Abstract

**Background:**

Although traditional lipid parameters and coronary imaging techniques are valuable for cardiovascular disease (CVD) risk prediction, better diagnostic tests are still needed.

**Methods:**

In a prospective, observational study, 795 individuals had extensive cardiometabolic profiling, including emerging biomarkers, such as apolipoprotein E–containing HDL-cholesterol (ApoE-HDL-C). Coronary artery calcium (CAC) score was assessed in the entire cohort, and quantitative coronary computed tomography angiography (CCTA) characterization of total burden, noncalcified burden (NCB), and fibrous plaque burden (FB) was performed in a subcohort (*n* = 300) of patients stratified by concentration of ApoE-HDL-C. Total and HDL-containing apolipoprotein C-III (ApoC-III) were also measured.

**Results:**

Most patients had a clinical diagnosis of coronary artery disease (CAD) (*n* = 80.4% of 795), with mean age of 59 years, a majority being male (57%), and about half on statin treatment. The low ApoE-HDL-C group had more severe stenosis (11% vs. 2%, overall *P* < 0.001), with higher CAC as compared with high ApoE-HDL-C. On quantitative CCTA, the high ApoE-HDL-C group had lower NCB (β = –0.24, *P* = 0.0001), which tended to be significant in a fully adjusted model (β = –0.32, *P* = 0.001) and altered by ApoC-III in HDL levels. Low ApoE-HDL-C was significantly associated with LDL particle number (β = 0.31; *P* = 0.0001). Finally, when stratified by FB, ApoC-III in HDL showed a more robust predictive value of CAD over ApoE-HDL-C (AUC: 0.705, *P* = 0.0001) in a fully adjusted model.

**Conclusion:**

ApoE-containing HDL-C showed a significant association with early coronary plaque characteristics and is affected by the presence of ApoC-III, indicating that low ApoE-HDL-C and high ApoC-III may be important markers of CVD severity.

**Trial Registration:**

ClinicalTrials.gov: NCT01621594.

**Funding:**

This work was supported by the NHLBI at the NIH Intramural Research Program.

## Graphical abstract

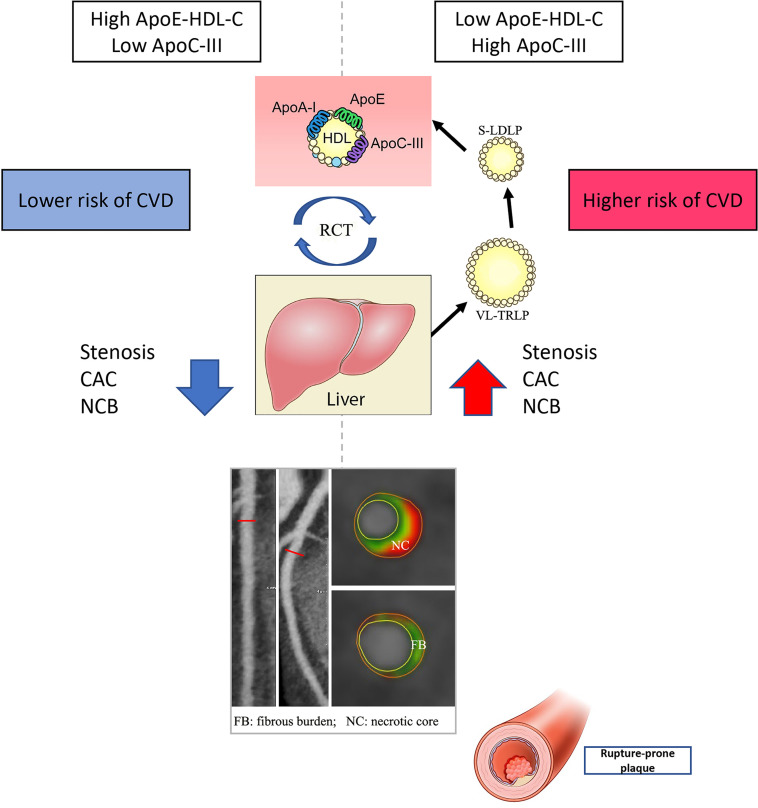

## Introduction

The measurement of plasma lipids and lipoproteins is a critical step in cardiovascular disease (CVD) risk assessment, but whether other related biomarkers could further improve risk prediction is of great interest for the routine management of cases. In regard to HDL-cholesterol (HDL-C), numerous clinical studies have established a strong inverse association between HDL-C and CVD (1, 2); however, recent epidemiological data suggest that the predictive value of HDL-C varies depending upon its blood concentration and inflammatory state. Low HDL-C serves as a biomarker of increased risk of all-cause mortality and nonfatal myocardial infarction even in statin-treated patients (3), whereas no further reduction in CVD risk was observed in patients with very high HDL-C values. In fact, patients with very high levels of HDL-C greater than 116 mg/dL in men and 135 mg/dL in women may have increased risk (4). Moreover, in statin-treated patients with very low LDL-C levels, residual cardiovascular risk is not predicted by HDL-C (5). This controversy, along with the failure of drugs that increase HDL-C to reduce CVD events (6), raises the question of whether other measures of HDL besides its cholesterol content (HDL-C), such as assays dependent on its function, might be more relevant (7). The most studied function of HDL is its role in reverse cholesterol transport (RCT), which can be assessed by measuring cholesterol efflux capacity (CEC; ref. 8). The function of HDL, however, has to be coupled in some way to its composition and disease specific pathophysiology. Hence, investigating the structural phenotype of HDL and related function under unique disease-determined states may open new fields of intervention and diagnostics.

HDL is a heterogenous lipoprotein, with apolipoprotein A-I (ApoA-I) being the main protein component. ApoE is also present on HDL but is far less abundant. These associated lipoproteins may alter the function of HDL and may therefore capture the relationship between HDL and CVD better than HDL-C alone (9). ApoE is known to play an important role in modulating cholesterol efflux (10) and in affecting the metabolism of ApoB-containing lipoproteins (11). Indeed, recent clinical studies revealed that low total plasma levels of ApoE are associated with higher risk of CVD (9, 12). It is believed that the major ApoE effects on lipoprotein metabolism are determined by its interaction with LDL and VLDL receptors, which promotes the hepatic clearance of lipoproteins (11). This interaction is modulated by proinflammatory ApoC-III, which is found on both HDL and triglyceride-rich lipoproteins (TRLPs). A rise in plasma triglycerides (TGs) under high ApoC-III levels accelerates the catabolism of HDL and inhibits the clearance of TRLPs from the plasma (13). Thus, the presence of ApoC-III along with ApoE in HDL may adversely affect the antiatherogenic properties of HDL (14).

To address the current gaps in CVD biomarkers, we aimed to estimate atherosclerosis burden by coronary artery calcium (CAC) and determine presence of early coronary artery disease (CAD) by applying quantitative coronary computed tomography angiography (CCTA) for measurement of coronary plaque parameters, including noncalcified plaque burden (NCB) and fibrous plaque burden (FB; ref. 15). Because of the high negative predictive value of CCTA for CAD risk stratification (16), we examined 2 potentially novel lipoprotein-related tests that could be implemented for routine testing, namely ApoE-containing HDL-C (ApoE-HDL-C) and the content of ApoC-III in HDL, for their association with CAC and CCTA.

## Results

### Study characteristics of the CVD cohort.

Enrolled patients with known CVD (*n* = 795) were mostly middle-aged (58.70 ± 13.53 years), predominantly White and male (*n* = 452; 56.9%), and overweight to obese (mean BMI ± SD: 28.92 ± 6.78), with almost half the cohort on statin treatment (47%) (Supplemental Table 1; supplemental material available online with this article; https://doi.org/10.1172/jci.insight.159577DS1). Some of the patients had history of percutaneous coronary intervention (PCI) (*n* = 204; 25.7%) and coronary artery bypass graft (CABG) surgery (*n* = 64; 8.1%) before study enrollment. The mean ApoE-HDL-C levels were 4.84 ± 1.76 mg/dL, consistent with previous reports (17).

Demographic and clinical characteristics of different ApoE-HDL-C subgroups (low, medium, and high) are shown in Table 1. We found higher statin treatment prevalence in low ApoE-HDL-C patients and concurrently lower total cholesterol (TC), LDL-C, and ApoB, as compared with high ApoE-HDL-C counterparts. Additionally, HDL-C and ApoA-I were significantly lower in the ApoE-HDL-C low group, whereas TG levels were higher (Table 1). Patients in the low ApoE-HDL-C group had more significant coronary artery stenosis classified by Coronary Artery Disease Reporting and Data System (CAD-RADS) as 4A (severe stenosis, 11% vs. 2%) and 5 (total occlusion, 10% vs. 3%) as compared with the high ApoE-HDL-C group (overall *P* < 0.001). Consistently, we found an inverse association between ApoE-HDL-C levels and stenosis severity in the entire cohort (Rho: –0.17, *P* = 0.03). Additionally, CAC score tended to be higher in the low ApoE-HDL-C group as compared with the high ApoE-HDL-C group (431.28 ± 957.05 vs. 235.33 ± 678.14, *P* = 0.02; Table 1); however, no significant association between ApoE-HDL-C levels and CAC score in the entire cohort was observed (Rho: –0.07, *P* = 0.13).

Despite decreased LDL-C in the low ApoE-HDL-C group, corresponding NMR analysis revealed significantly increased S-LDLP number and TRLPs as compared with patients in the high ApoE-HDL-C group (Table 1). The rest of the NMR analysis showed positive association of total LDLP number with low ApoE-HDL-C (β = 0.31, *P* < 0.0001; Supplemental Table 2).

### Study characteristics of the CCTA subcohort.

Next, we created a CCTA subcohort (*n* = 300) to test the relationship between ApoE-HDL-C concentration and coronary artery burden. We also hypothesized that inclusion of ApoC-III measurement in the analysis might improve its proposed association with CVD as stated before (18). For this subcohort, patients with high and low ApoE-HDL-C levels were identified based on the cut points from the original CVD cohort, whereas ApoC-III groups’ definition was based on the measured ApoC-III levels in HDL and cut points from this CCTA subcohort.

Overall, the CCTA subcohort was representative of the CVD cohort in terms of demographic and clinical characteristics. Demographic and clinical characteristics of the ApoE-HDL-C subgroups (low and high) are shown in Table 2. Both low and high ApoE-HDL-C subgroups were matched by age and BMI, mostly represented middle-aged and overweight men, and tended to be on statin treatment in a slightly higher rate as compared with the high ApoE-HDL-C subgroup (Table 2). As expected, the low ApoE-HDL-C group had higher hsCRP levels, 3.26 mg/L (IQR 0.7–4.5) versus 2.83 mg/L (IQR 0.6–2.3), *P* = 0.02, than in high ApoE-HDL-C patients. Moreover, high ApoE-HDL-C levels were negatively associated with hsCRP (β = –0.17, *P* < 0.0001). Of note, ApoC-III in HDL levels followed the same pattern of changes as seen in the ApoE-HDL-C low and high subgroups (Table 2) and corresponded to the values reported in a CVD cohort (14). Similar to the whole cohort, subjects in the low ApoE-HDL-C group had more coronary artery stenosis classified by CAD-RADS as 4A (severe stenosis) and 5 (total occlusion) as compared with the high ApoE-HDL-C group (overall *P* = 0.01). Additionally, CAC score tended to be higher in the low ApoE-HDL-C group as compared with the high ApoE-HDL-C (239.39 ± 526.55 vs. 111.33 ± 288.29, *P* = 0.01).

Table 3 and Table 4 show the association between CCTA plaque characteristics and ApoE-HDL-C over HDL-C. In the high ApoE-HDL-C stratum and in the entire subcohort, ApoE-HDL-C level correlated with TB and NCB and had similar strength of association with TB and NCB as HDL-C and ApoA-I (Table 3). Interestingly, CAC score showed a more significant correlation with total ApoE-HDL-C levels than HDL-C or ApoA-I. Further stratification with the addition of ApoC-III in HDL-C resulted in notably different association with CCTA plaque parameters (Table 4). High ApoE and high ApoC-III in HDL-C negatively correlated with TB (β = –0.20; *P* = 0.001) and NCB (β = –0.21; *P* = 0.0001). In contrast, lower concentration of ApoC-III in HDL-C and high ApoE resulted in more robust negative association with the same plaque parameters (β = –0.27; *P* = 0.0001 for both). The relationship of TB and NCB with high ApoE-HDL-C persisted in fully adjusted analyses (β = –0.31 and β = –0.32 correspondingly, *P* = 0.001), whereas low ApoE-HDL-C showed a positive association with NCB (β = 0.13; *P* = 0.02), which became insignificant after hsCRP adjustment (Supplemental Table 3). Of note, high ApoE with high ApoC-III in HDL-C group was characterized by lower TB and NCB levels as compared with low ApoC-III in HDL-C (*P* = 0.01), whereas plaque morphology characteristics remained increased in the low ApoE-HDL-C group independent of ApoC-III in HDL-C levels (Supplemental Table 4). Indeed, comparison of TB and NCB between high ApoC-III subgroups based on ApoE-HDL-C levels showed a statistically significant difference (0.94 ± 0.43 vs. 1.15 ± 0.40, *P* = 0.002; 0.90 ± 0.42 vs. 1.08 ± 0.37, *P* = 0.01, respectively).

**Figure 1:**
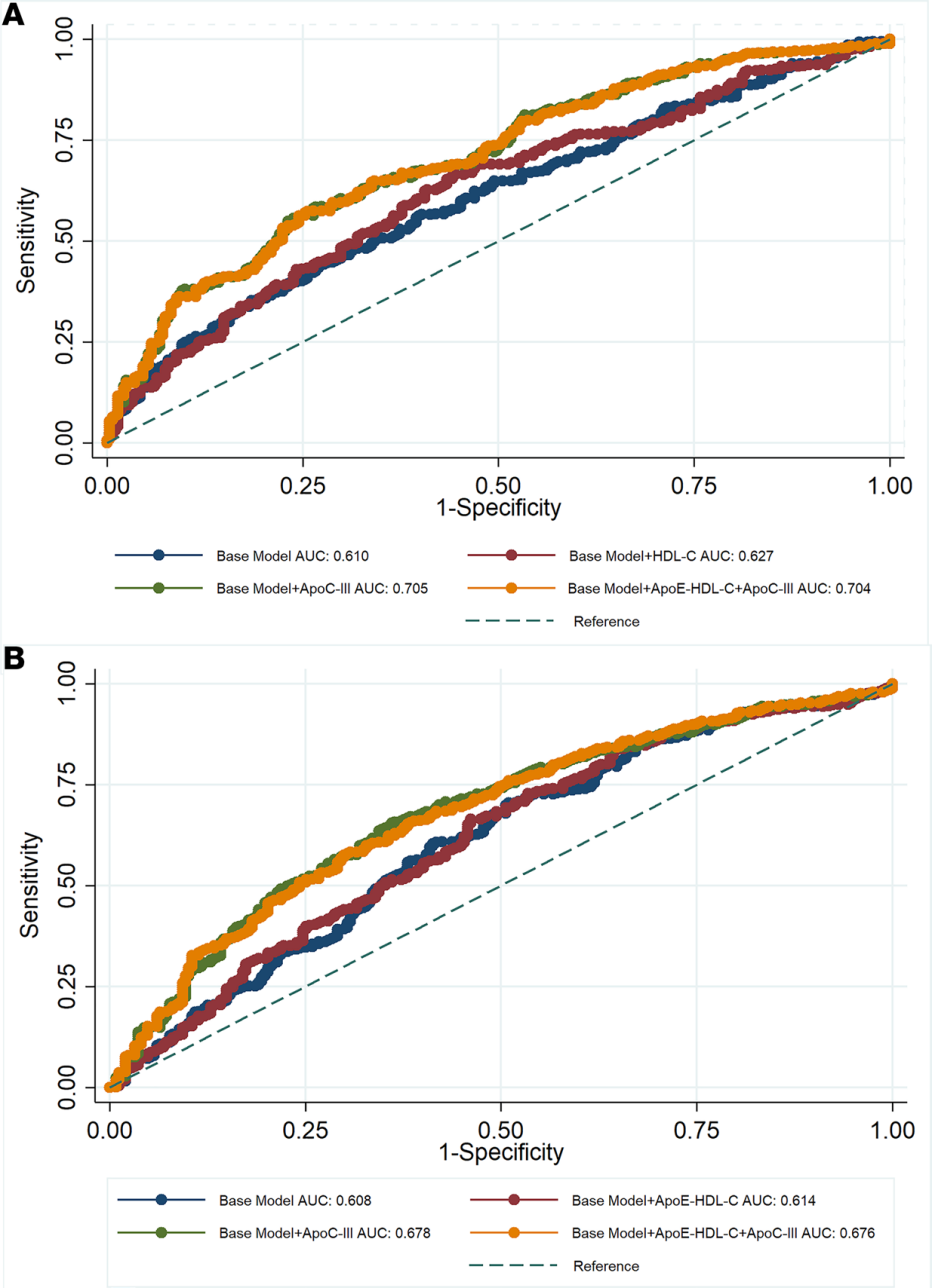
Results comparing logistic regression models with AUC ROCs were reported. Pearson’s χ^2^ test was applied for estimating *P* values with significance level of *P* < 0.05. Predicting of CAD based on CCTA plaque parameters in (**A**) fibrous plaque burden (*P* = 0.0001) and (**B**) necrotic burden (*P* = 0.01). Base model includes age, sex, current smoking, BMI, statin treatment, hsCRP, LDL-C, and TGs. Hs(CRP), high-sensitivity C-reactive protein.

Further analyses of vulnerable plaque parameters revealed significant positive associations of fibrous, fibro-fatty, and necrotic burden with combined low ApoE-HDL-C and high ApoC-III in HDL concentrations (β = 0.55, β = 0.52, β = 0.37, *P* = 0.0001 for all, respectively; Table 4). The opposite relationship was established for high ApoE-HDL-C and high ApoC-III in HDL group. To better explore the observed association and discrepancies in plaque morphology characteristics, as well as contribution of ApoC-III in assessing plaque vulnerability parameters beyond traditional CVD risk factors, receiver operating characteristic (ROC) analysis was performed. The combination of ApoC-III with ApoE-HDL-C resulted in better predictive values for the FB when added to the base model adjusted for traditional risk factors, including statin treatment and hsCRP (AUC for base model with ApoE-HDL-C+ApoC-III: 0.704, 95% CI 0.668–0.741; AUC for base model with ApoE-HDL-C: 0.627, 95% CI 0.587–0.667; *P* = 0.0001; Figure 1A). However, ApoC-III alone had similar predictive value (AUC: 0.705) as in combination with ApoE-HDL-C over ApoE-HDL-C and HDL-C (AUC: 0.627), suggesting its predominant role in the described complex association between CAD and HDL. Similar results were observed in predicting necrotic plaque component (AUC, 95% CI: 0.678, 0.635–0.716 vs. 0.614, 0.572–0.657; *P* = 0.01) (Figure 1B). Finally, no significant differences were detected in predicting CAC (*P* = 0.05). Interestingly, combination of high ApoE-HDL-C with low ApoC-III in HDL concentration resulted in the most significant decrease of all 3 plaque morphology index characteristics, and lower levels of these measurements were retained in low ApoE-HDL-C and low ApoC-III groups (Supplemental Table 4).

We next sought to determine if this complex association of ApoE-HDL-C with ApoC-III can be explained by difference in lipoprotein lipase (LpL) activity. To study how effectively LpL hydrolyzes lipoproteins in plasma samples with high ApoC-III/low ApoE-HDL-C levels compared to plasma samples with low ApoC-III/high ApoE-HDL-C levels, selected plasma samples were spiked with purified LpL and released fatty acid (FA) was quantified. Unexpectedly, low total ApoC-III levels resulted in overall lower LpL activity as compared with high total ApoC-III levels (5.02 ± 2.27 nmol FA released per hour versus 5.98 ± 2.08 nmol FA released per hour, *P* = 0.03); however, no significant differences were observed between the compared groups for the TRLPs (Supplemental Table 5). Instead, low ApoE-HDL-C with high total plasma ApoC-III tended to have an increased FB and higher CAC score and S-LDLP (*P* = 0.01 for both), which support the observed CCTA results. Finally, LpL activity was positively correlated with FB in both low (β = 0.34; *P* = 0.0001; β = 0.46; *P* = 0.0001, fully adjusted) and high (β = 0.22; *P* = 0.02; β = 0.17; *P* = 0.08, fully adjusted) total ApoC-III subgroups (Table 5).

NMR lipid analysis revealed positive association with L-LDLP in the high ApoE-HDL-C group (β = 0.35; *P* = 0.0001), whereas M-LDLP was contributing to low ApoE-HDL-C (β = 0.32; *P* = 0.0001). Last, high ApoE-HDL-C was positively correlated with L-HDLP (β = 0.69; *P* = 0.0001) and negatively with S-LDLP (β = –0.23; *P* = 0.01), while S-HDLP contributed to the low ApoE-HDL-C group (β = 0.32; *P* = 0.0001) (Supplemental Table 6).

## Discussion

Findings from this observational study suggest that low levels of ApoE-HDL-C are characterized by an unfavorable cardiometabolic phenotype and strongly associated with early coronary plaque development as assessed by CCTA. Presence of ApoC-III in HDL alone and in conjunction with ApoE-HDL-C suggests that ApoC-III might be the main driving force of the described association between CAD and HDL, as well as improving the predictive value of some coronary plaque characteristics over traditional CVD risk factors.

During the last several decades there have been major advances in CVD prevention and treatment; however, the CVD death rate remains high (19). One possible area of future improvement relates to diagnostic testing in order to better identify people who would benefit most from therapy. HDL-C is routinely used for primary CVD risk stratification (2), though recent studies have revealed contradictory findings in regard to the role of HDL in the pathogenesis of CVD (20), prompting the development of novel markers like those related to its function, such as CEC (21). Indeed, there are now multiple studies demonstrating the utility of CEC in CVD risk stratification (22, 23). One of the possible mechanisms attributable to RCT dysfunction relates to ApoE, an exchangeable apolipoprotein, with major roles in mediating the interaction of circulating lipoproteins with tissues by binding to membrane receptors (24, 25). Increases in both ApoE and phospholipid transfer protein activity have been shown to improve the delivery of energy substrates and phospholipids to tissues for sustaining cellular membrane homeostasis in patients with systemic inflammatory response (25). Furthermore, a genome-wide association study identified common genetic variation at the APOE locus as a significant determinant of CEC independent of HDL-C (26). Moreover, ApoE-containing HDL particles promote cholesterol efflux from extrahepatic cells (10) by ABCA1- and ABCG1-dependent processes, and this process is antagonized by the presence of ApoC-III (9). Finally, it has been shown that cognitive decline might be dependent on the ApoE-ε4 genotype and sex through FA metabolism and related metabolic pathways (27).

Interestingly, a recent study reported that ApoE-HDL lacking ApoC-III was associated with better cognitive function and lower dementia risk (28). The cumulative evidence supports a complex biologic interaction between ApoE and ApoC-III in HDL function and how it relates to CVD. Our current observations illustrate that people with CVD with optimal total and LDL-C under statin treatment had low HDL-C and high TGs potentially determined by ApoE/ApoC-III ratio in HDL, which is in line with previous results (12). It is known that low HDL-C is correlated with elevated serum TG and remnant lipoproteins and is strongly and inversely associated with CVD risk (29). In agreement with prior investigations (30), HDLP size expansion by the addition of ApoE to HDL was observed in the high ApoE-HDL-C group and related to L-HDLPs, whereas low ApoE-HDL-C associated with S-HDLPs or M-LDLPs and increased TRLPs. Our analysis shows a clinically worse cardiometabolic profile, more significant coronary artery stenosis, and higher CAC scores in patients with low ApoE-HDL-C as compared with the high ApoE-HDL-C group. Interestingly, prior studies suggest that total plasma ApoE may be related to a beneficial effect on the arterial wall. In particular, the cardioprotective effect of ApoE on arterial stiffness may be mediated by COX-2 and miR-145, which are known to be involved in inflammatory regulation (30).

Considering the above results and potential effects of statin treatment on the detected observations, we further investigated the relation of ApoE-containing HDL-C and residual CVD risk. For this purpose, a reliable, noninvasive imaging technique, quantitative CCTA, was applied for the subcohort of participants with CAD. Prior work has demonstrated that noncalcified plaque (31) and its morphological characteristics (32) predict prospective cardiovascular events beyond traditional CVD risk factors and statin treatment. The observed inverse association of high ApoE-HDL-C with NCB in multivariable linear regression models and increased levels of TB and NCB in the low ApoE-HDL-C group suggest a potentially unique role of ApoE in coronary plaque estimation over traditional lipid constituents of HDL.

To explore the observed differences based on high or low ApoE-HDL-C, we further screened our CCTA subcohort for the presence of ApoC-III. Interestingly, high ApoE-HDL-C with high ApoC-III in HDL was negatively associated with less stable plaque based on NCB and plaque morphology parameters, such as fibrous and necrotic burden. Moreover, the observed association was characterized by lower levels of NCB under high ApoE-HDL-C concentration affected by the presence of ApoC-III. In both low and high ApoE-HDL-C groups, only the high ApoC-III subgroup contributed to the increased vulnerable plaque morphological characteristics.

While these associations have been detected in a limited number of patients, it represents an important observation that may provide new insights into our current understanding of the complex interrelationship between ApoE and ApoC-III. Our results are also consistent with previous studies showing an atheroprotective role of ApoE-HDL (33) and the opposite interaction with ApoC-III, which contributes to coronary artery calcification and subclinical atherosclerosis (18). It has been reported before that ApoC-III may further alter HDL composition (34), and its concurrent presence with ApoE in HDL abrogates protective HDL function and increases CVD risk (14).

To elaborate more on the CVD risk estimation, we further explored the ApoE and ApoC-III in HDL predictive value based on contribution to coronary plaque compositional phenotype. As a result, ApoC-III in HDL alone showed a better predictive value for CAD than HDL-C or ApoE-HDL-C for FB and necrotic component even after adjusting for statin treatment and hsCRP. Interestingly, combination of ApoC-III in HDL with ApoE-HDL-C over ApoE-HDL-C revealed the predominant role of ApoC-III in the observed association with CAD. The potential mechanism behind this might be related to an unfavorable HDL apolipoprotein content characterized by low ApoE and high ApoC-III ratio, which in turn decreases RCT, activates LpL and subsequent plasma TG increase, and alters composition of retained LDL in the arterial wall and initiates inflammatory and proatherogenic processes (35).

Indeed, we observed a significant increase in S-LDLP and very small TRLPs in the ApoE-HDL-C low group, which was consistent among CVD and CCTA cohorts. It is known that high levels of ApoC-III possess inhibitory effects on LpL and TRLP uptake, which can be altered by the ApoE-HDL presence (9, 36) Thus, we hypothesized that high ApoE-HDL-C with low total plasma ApoC-III subgroup would result in higher LpL activity as compared with low ApoE-HDL-C with high total plasma ApoC-III. Although we were unable to show the direct effect of total ApoC-III levels in the presence of ApoE-HDL-C on LpL activity, because LpL activity is almost negligible in plasma before heparin injection (37), there might be some effects of LpL on coronary plaque composition mediated through LDL metabolism and impaired TRLP hepatic clearance. In our LpL subgroup analyses, high LpL activity under low ApoE-HDL-C and high total ApoC-III did not have a strong association with TG metabolism but was associated with a significantly higher S-LDLP concentration. Moreover, our observations are supported by the previous results finding that ApoC-III mainly increases plasma TGs by inhibition of lipoprotein uptake by LDL receptors (38, 39), followed by generation of smaller and dense LDL particles, which are highly susceptible to oxidation and atheroprone (40). Also, the observed findings might be related to sex hormone differences reported in animal species, which are known to affect both ApoE (41) and LpL function (42). Indeed, sex differences are also well clinically documented, and men tend to have higher risk of CVD development (43). The sex-specific effects attributed to the ApoE/ApoC-III associations we observed in this study warrant future research.

There are several strengths and limitations to the current study worth noting. To our knowledge, this is the first study to investigate the relationship between different levels of ApoE-HDL-C, along with further ApoC-III on CVD progression as determined by CAC and CCTA. Furthermore, potential main confounders, such as BMI and statin treatment, were adjusted in multivariate modeling and were matched in the subgroup analyses. Prospective larger CVD cohort studies would be valuable for more carefully assessing the clinical utility of ApoE-HDL-C and ApoC-III in predicting early CVD, especially in populations of different sexes and races. Also, a minor portion of the CCTA subcohort patients in our study had other CVD abnormalities, although this number was relatively low to affect the established overall study assumptions. Finally, more mechanistic studies are also needed for investigating disease-specific and complex biological effects of ApoE/ApoC-III lipoprotein transport and metabolism interactions.

In conclusion, we demonstrate for the first time to our knowledge that ApoE-HDL-C along with ApoC-III in HDL may predict CVD severity as determined by CCTA. Furthermore, we also show that low ApoE-HDL-C in conjunction with high ApoC-III associate with early high-risk soft coronary plaque as assessed by NCB and FB. Hence, the potentially novel biomarkers reported in this study should be further examined in larger CVD and other cohorts for their clinical utility in estimating CVD risk.

## Methods

### Study design and overview.

**Figure 2:**
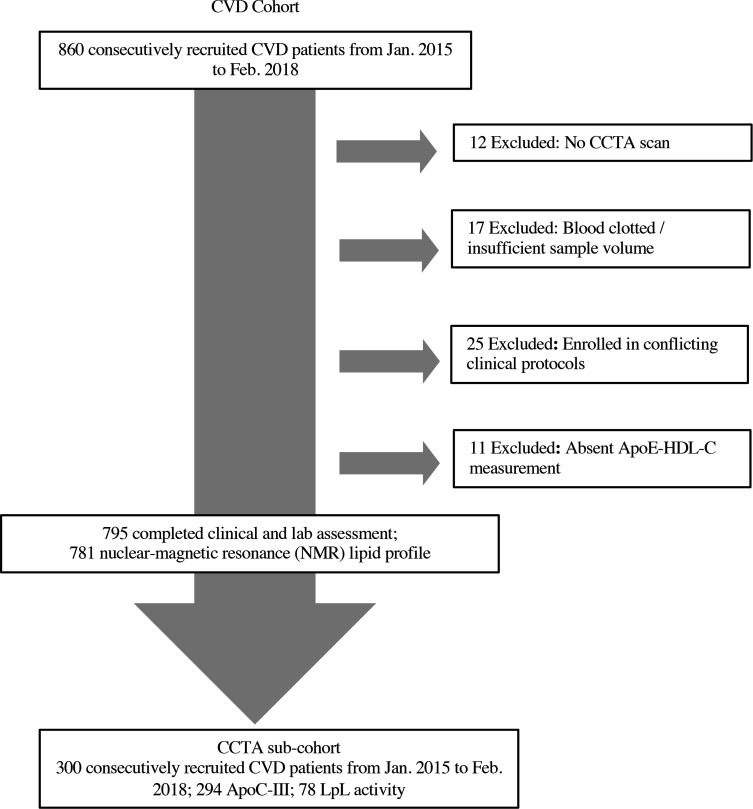
Recruitment and follow-up scheme of study participants.

A total of 860 patients with known CVD were recruited from January 2015 through February 2018 as a part of an ongoing, prospective, observational study (PREDICT, Prospective Evaluation of New Techniques in Radiation Reduction for Cardiovascular Computed Tomographic Angiography) for evaluating new cardiovascular imaging techniques. Here, data analyses were done in 795 consecutive patients with diagnosed CAD who completed clinical assessment and met inclusion criteria (Figure 2). Patients were excluded if they were pregnant or had severe renal disease (EGFR < 30 mL/min/1.73 m^2^). A complete list of inclusion/exclusion criteria can be found at ClinicalTrials.gov, NCT01621594. All patients had CCTA and CAC score assessment. Retrospective deidentified demographic, clinical, and laboratory data were retrieved from medical records through the NIH clinical research repository, Biomedical Translational Research Information System (BTRIS). A subcohort of 300 CVD patients were selected based on their ApoE-HDL-C levels (see below) and underwent additional quantitative CCTA coronary artery characterization.

Data that support the findings of this study are available to qualified researchers trained in human patient confidentiality protocols from the corresponding author upon request. Strengthening the Reporting of Observational Studies in Epidemiology guidelines were followed for reporting the findings (44).

### Biochemical measurements.

Peripheral blood from the enrolled patients was collected in EDTA-coated tubes after overnight fasting and centrifuged for 20 minutes at 3500 rpm at 4°C. Obtained plasma was aliquoted and immediately stored at –80°C until further analysis without being exposed to a freeze-thaw cycle. Traditional plasma lipid parameters included TC, HDL-C, and TG levels, which were measured using commercially available enzymatic methods on the Cobas 6000 analyzer (Roche Diagnostics). LDL-C was calculated by the Friedewald equation. ApoA-I and ApoB concentrations were measured by automated turbidometric immunoassays on the Cobas 6000 analyzer. Other plasma biochemical measurements, including hsCRP, were performed on a Cobas 6000 analyzer in the NIH Clinical Center.

In addition, we used homogenous assays (Denka Seiken Co, Ltd) for measuring direct HDL-C/LDL-C, ApoE-HDL-C, LDL-TG, and sdLDL-C as described previously (45). Briefly, by using specific surfactants exhibiting different reactivity, total HDL-C was fractionally assayed as ApoE-HDL-C and ApoE-deficient HDL-C. ApoE-HDL-C/HDL-C ratio was generated based on the Denka lipid parameters.

ApoC-III was measured by the quantitative commercially available ELISA kit from AssayPro in both whole and ApoB-depleted plasma. LipoSep IP (Sun Diagnostics) was used for ApoB depletion as described previously (46).

LpL activity was determined by combining plasma samples at final TG concentration of 12 mg/dL with a total of 0.6 units of purified LpL (L2254; MilliporeSigma) in PBS (pH 7.4) with 10 USP U/mL heparin (Fresenius Kabi). Additionally, 2 mg/mL FA-free bovine serum albumin (ICN Biomedicals) was added to the samples. Reaction mixtures were incubated on ice for 30 minutes and thereafter at 37°C for 1 hour. LpL activity was quantified by measuring released FA by nonesterified FA kit (FUJIFILM Wako Diagnostics) on a Synergy H1 microplate reader (BioTek) as previously described (47).

To further assess lipoprotein subclass profiles along with GlycA, we used the automated Vantera clinical NMR analyzer (Labcorp). The LipoProfile-4 algorithm was used to measure the following lipoprotein subclass parameters: VLDLP size (VLDL-Z) and number (VLDLP), TRLP, and the following subfractions: very small, small, medium, and large TRLP; LDLP sizes (LDL-Z) and number (LDLP), as well as their subfractions: small, medium, and large LDLP; HDLP size (HDL-Z) and number (HDLP), as well as their subfractions: small HDLP (HDL-P1~2), medium HDLP (HDL-P3~4), and large HDLP (HDL-P5~7).

### Coronary artery imaging.

All participants underwent CCTA on the same day as the blood draw, using the same CT scanner (320-detector row Aquilion ONE ViSION). Radiation exposure was in accordance with the NIH Radiation Exposure Committee guidelines. CCTA scan evaluation was done based on the CAD-RADS classification (48). Severe CAD was defined as total coronary occlusion (CAD-RADS 5), and non-CAD was defined as no significant stenosis or minimum stenosis (CAD-RADS 0 or CAD-RADS 1). All scans were initially reviewed for quality and presence of artifacts, thus precluding a reliable qualitative and quantitative evaluation. Coronary artery burden adjusted for luminal attenuation was evaluated across each of the 3 main coronary arteries by means of semiautomated software: QAngio CT (Medis; ref. 49). Manual adjustment of inner lumen and outer vessel wall delineations was performed if needed. TB, NCB, and DCB indices (mm^2^) were calculated by dividing total vessel plaque volume by total vessel length. TB was defined as the sum of calcified plaque burden and NCB. Noncalcified plaque subcomponents including fibrous, fibro-fatty, and necrotic burdens were obtained after adaptively correcting for lumen attenuation and depicted based on Hounsfield units.

CAC was evaluated as a part of the normal workflow by an experienced cardiologist, using semiautomated software (SmartScore, GE Healthcare). CAC (mean total Agatston scores) was measured using electron beam tomography from 40 continuous 3 mm thick computed tomograms (Imatron). A single experienced radiological technologist performed scoring, following a protocol blinded to clinical and laboratory data, using customized software (Imatron). Natural log transformation of CAC scores, (ln[CAC + 1]), was performed to account for the high percentage of CAC scores of 0 in all groups (50).

### Statistics.

Data are presented as the mean ± SD for parametric variables or the median (IQR) for nonparametric variables and as number (%) for categorical variables. Skewness and kurtosis measures were considered to assess normality. Non-normally distributed data were log-transformed to account for non-Gaussian distributions. One-way ANOVA for continuous variables and Pearson’s χ^2^ test for categorical variables were used to compare the distribution between different ApoE-HDL-C groups. Intergroup comparison was done by Student’s 2-tailed *t* test for parametric variables and Wilcoxon’s rank-sum test for nonparametric variables. Spearman’s correlation testing and univariable linear regression analyses were performed to assess the potential relationship between quantitative CCTA plaque characteristics and levels of ApoE-HDL-C or ApoC-III. We modeled ApoE-HDL-C both as a continuous and a categorical variable. The cut points for the categorical variable were obtained from the distribution of ApoE-HDL-C in the whole analysis population of 795 patients (LOW: <25th, MED: 25th–75th, and HIGH: >75th percentile values were <3.7 mg/dL, 3.7–5.7 mg/dL, and >5.7 mg/dL, respectively).

To estimate the prognostic value of ApoE-HDL-C and ApoC-III on coronary artery burden parameters, we created a subcohort of 300 patients based on the categorical ApoE-HDL-C cut points derived from the whole CVD cohort (LOW: <50th and HIGH: >50th percentile values were <4.6 mg/dL for low and >4.6 mg/dL for high). Additional ApoC-III subgroups were formed from the cut points obtained from the distribution of ApoC-III in HDL in the same CCTA subcohort of 300 patients (LOW: <50th and HIGH: >50th percentile values were <3.30 mg/dL for low and >3.30 mg/dL for high). LpL activity subgroups were made based on the cut points obtained from the distribution of total plasma ApoC-III in the same CCTA subcohort (LOW: <50th and HIGH: >50th percentile values were <11.42 mg/dL and >11.42 mg/dL, respectively). In these subgroups, 78 plasma samples were paired based on the TG concentration in each pair that did not differ more than 4 mg/dL. In order to justify the sample size in our subcohort to derive associations between ApoE-HDL-C and CCTA coronary plaque parameters, we calculated that addition of ApoE-HDL-C would augment the adjusted *R*^2^ value by at least 5% in linear regression models. Based on this assumption, our sample size was sufficient to have more than 90% power to detect significant associations (*P* < 0.05). In the utilized multivariable regression analysis models, we adjusted for covariates by including traditional cardiovascular risk factors, such as age, sex, current smoking, BMI, statin treatment, hsCRP, LDL-C, HDL-C, and TGs. Standardized β-coefficient values along with *P* values were reported for these analyses.

To further estimate the prognostic value of ApoC-III in addition to ApoE-HDL-C levels on coronary artery burden, ROC curve analyses were conducted after dichotomizing CCTA plaque characteristics based on median values. Data were represented as AUC with the 95% CI. Multivariable logistic regression analyses were used to compare the AUC for base model to models with HDL-C, ApoE-HDL-C, ApoC-III, and ApoE-HDL-C + ApoC-III. The base model was adjusted for age, sex, current smoking, BMI, statin treatment, hsCRP, LDL-C, and TGs. We applied a Bonferroni-corrected threshold to determine statistical significance in each analysis as described in the Results section and in the tables. Analysis was performed using Stata/IC 12.1 (StataCorp LP).

### Study approval.

Study approval was granted by the NIH NHLBI institutional review board in keeping with the Declaration of Helsinki. All study participants submitted written informed consent prior to enrollment.

## Author contributions

AVS, NNM, and ATR had full access to all the data in the study and take responsibility for the integrity of the data and the accuracy of the data analysis. AVS, MYC, NNM, and ATR conceived the study concept and designed the study. AVS, NP, KMA, CL, MR, GG, M Sampson, and MPP acquired and analyzed the data. AVS drafted the manuscript. AVS, NP, KMA, CL, MR, GG, M Sampson, MPP, AKD, AR, HLT, M Stagliano, MA, MYC, NNM, and ATR provided critical revisions of the manuscript. AVS, AR, and AKD performed statistical analyses. NNM and ATR provided technical guidance to AVS during the study. The study was conducted under the supervision of NNM and ATR.

## Supplementary Material

ICMJE disclosure forms

## Figures and Tables

**Table 1:**
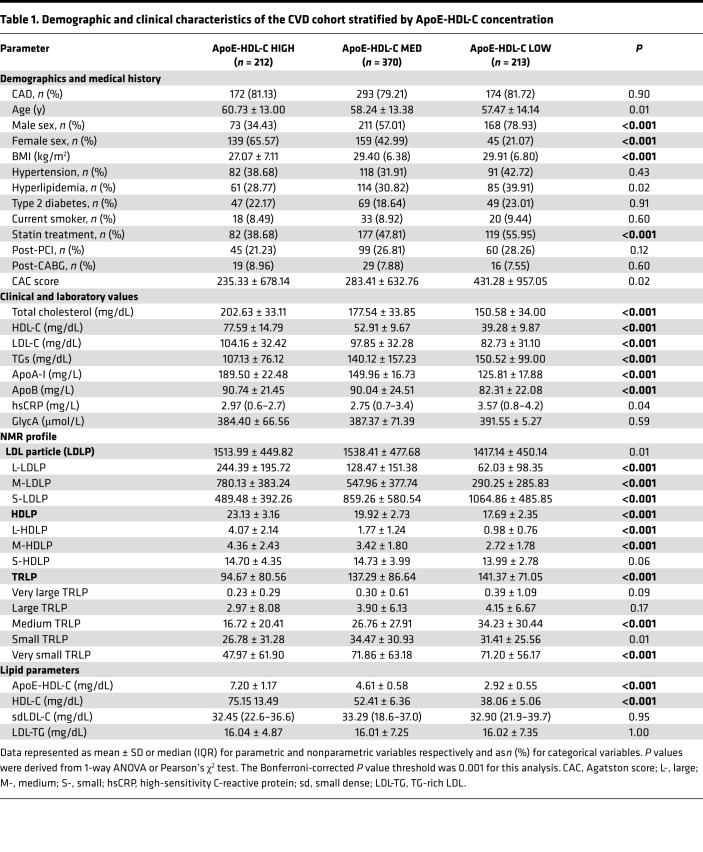
Demographic and clinical characteristics of the CVD cohort stratified by ApoE-HDL-C concentration

**Table 2:**
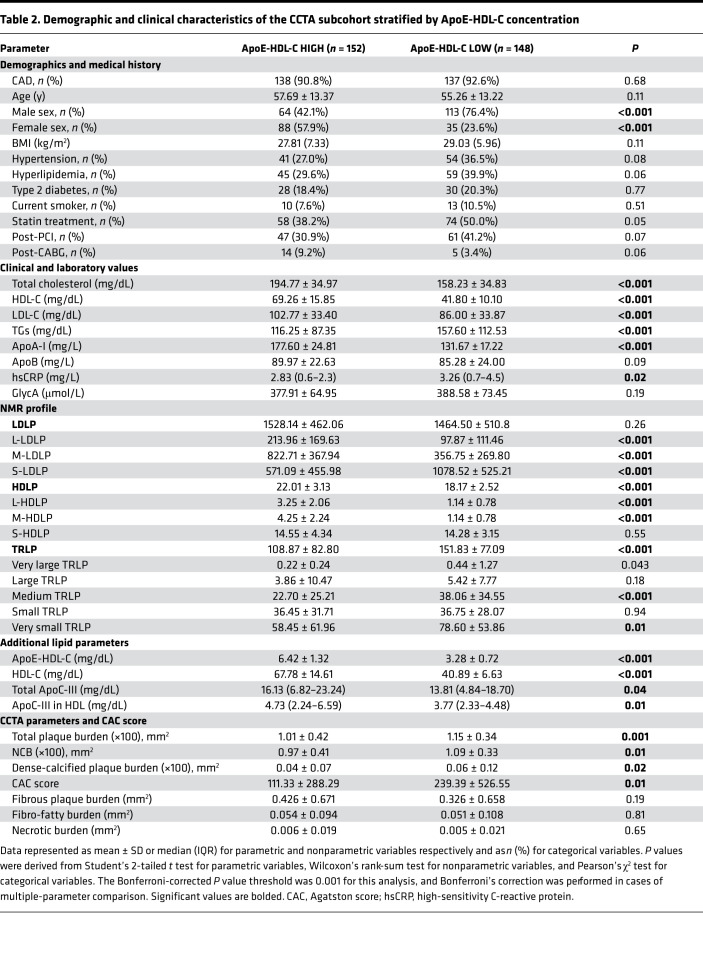
Demographic and clinical characteristics of the CCTA subcohort stratified by ApoE-HDL-C concentration

**Table 3:**
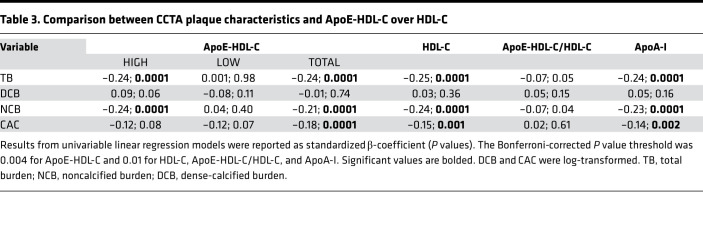
Comparison between CCTA plaque characteristics and ApoE-HDL-C over HDL-C

**Table 4:**
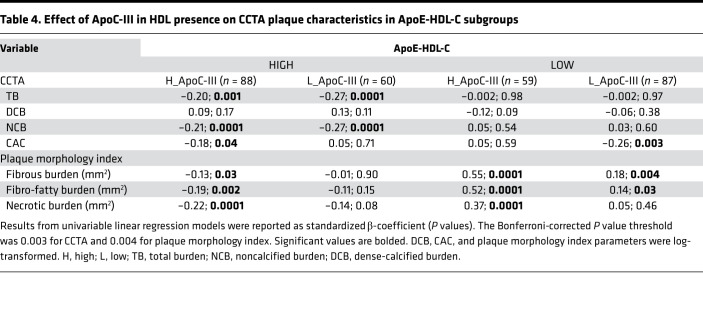
Effect of ApoC-III in HDL presence on CCTA plaque characteristics in ApoE-HDL-C subgroups

**Table 5:**
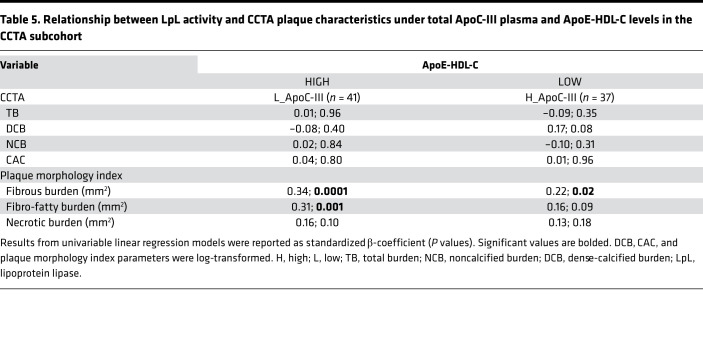
Relationship between LpL activity and CCTA plaque characteristics under total ApoC-III plasma and ApoE-HDL-C levels in the CCTA subcohort

## Version 1 04/07/2022

In-Press Preview

## Version 2 05/23/2022

Electronic publication
